# *N*′-[1-(2-Hydr­oxy-5-methyl­phen­yl)ethyl­idene]benzene­sulfonohydrazide

**DOI:** 10.1107/S1600536808025932

**Published:** 2008-08-16

**Authors:** Musalem Laila, Hapipah M. Ali, Subramaniam Puvaneswary, Ward T. Robinson, Seik Weng Ng

**Affiliations:** a Department of Chemistry, University of Malaya, 50603 Kuala Lumpur, Malaysia

## Abstract

The two independent mol­ecules in the asymmetric unit of the title compound, C_15_H_16_N_2_O_3_S, are each linked by an N—H⋯O_sulfon­yl_ hydrogen bond into a linear chain that runs along the shortest axis of the triclinic unit cell. The hydr­oxy groups are engaged in intra­molecular hydrogen bonding and the amino N atom shows pyramidal coordination.

## Related literature

For 2′-(2-hydroxy­phenyl-1-ethyl­idene)benzene­sulfono­hydrazide, which adopts a hydrogen-bonded chain structure, see: Ali *et al.* (2007 ▶).
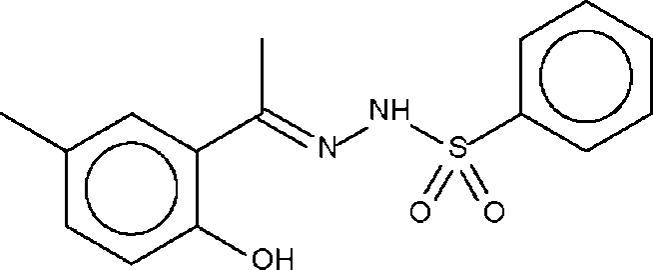


## Experimental

### 

#### Crystal data

C_15_H_16_N_2_O_3_S
*M* *_r_* = 304.36
Triclinic, 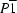

*a* = 5.1547 (1) Å
*b* = 17.0321 (2) Å
*c* = 18.2635 (1) Å
α = 63.192 (1)°
β = 88.577 (1)°
γ = 86.345 (1)°
*V* = 1428.19 (4) Å^3^
*Z* = 4
Mo *K*α radiation
μ = 0.24 mm^−1^
*T* = 100 (2) K
0.18 × 0.14 × 0.06 mm

#### Data collection

Bruker SMART APEX diffractometer
Absorption correction: multi-scan (*SADABS*; Sheldrick, 1996 ▶) *T*_min_ = 0.958, *T*_max_ = 0.986
12657 measured reflections
6415 independent reflections
5603 reflections with *I* > 2σ(*I*)
*R*_int_ = 0.018

#### Refinement

*R*[*F* ^2^ > 2σ(*F* ^2^)] = 0.042
*wR*(*F* ^2^) = 0.132
*S* = 1.04
6415 reflections
399 parameters
4 restraints
H atoms treated by a mixture of independent and constrained refinement
Δρ_max_ = 0.54 e Å^−3^
Δρ_min_ = −0.58 e Å^−3^

### 

Data collection: *APEX2* (Bruker, 2007 ▶); cell refinement: *SAINT* (Bruker, 2007 ▶); data reduction: *SAINT*; program(s) used to solve structure: *SHELXS97* (Sheldrick, 2008 ▶); program(s) used to refine structure: *SHELXL97* (Sheldrick, 2008 ▶); molecular graphics: *X-SEED* (Barbour, 2001 ▶); software used to prepare material for publication: *publCIF* (Westrip, 2008 ▶).

## Supplementary Material

Crystal structure: contains datablocks global, I. DOI: 10.1107/S1600536808025932/bq2092sup1.cif

Structure factors: contains datablocks I. DOI: 10.1107/S1600536808025932/bq2092Isup2.hkl

Additional supplementary materials:  crystallographic information; 3D view; checkCIF report

## Figures and Tables

**Table 1:** Hydrogen-bond geometry (Å, °)

*D*—H⋯*A*	*D*—H	H⋯*A*	*D*⋯*A*	*D*—H⋯*A*
O1—H1*o*⋯N1	0.84 (3)	1.80 (2)	2.562 (2)	151 (4)
O4—H4*o*⋯N3	0.85 (3)	1.79 (2)	2.563 (2)	150 (3)
N2—H2*n*⋯O2^i^	0.88 (1)	2.18 (1)	3.040 (2)	168 (2)
N4—H4*n*⋯O5^ii^	0.88 (1)	2.07 (1)	2.942 (2)	173 (2)

Symmetry codes: (i) 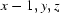; (ii) 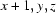.

**Fig. 1.**
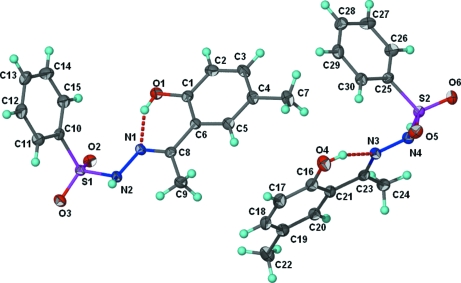
Thermal ellipsoid plot of the asymmetric unit of (I) (Barbour, 2001) at the 70% probability level. Dashed line indicates H-bonding.

## supplementary crystallographic information

### Comment

2'-(2-Hydroxyphenyl-1-ethylidene)benzenesulfonohydrazide adopts a hydrogen-bonded chain structure; the chain runs along the *a*-axis of the monoclinic unit cell and the repeat distance is the length of the this axis, *i.e.*, 5.18 Å (Ali *et al.*, 2007). An additional methyl group in the molecule (Scheme I) does not result in any significant difference in both structure and packing (Fig. 1). The two independent molecules are each linked by an *N*–*H*···O sulfonyl hydrogen-bond into a linear chain that runs along the shortest axis of the triclinic unit cell; the repeat distance is 5.15 Å. The hydroxy groups are engaged in intramolecular hydrogen bonding (Table 1.).

### Experimental

The Schiff base was prepared by refluxing by benzene sulfanohydrazide (0.40 g, 0.64 mmol) and 5-methyl-2-hydroxyacetophenone (0.10 g, 0.64 mmol) in ethanol for 2 h. The product was filtered and recrystallized from ethanol.

### Refinement

Carbon-bound hydrogen atoms were generated geometrically (C—H 0.95 to 98 Å), and were treated as riding, with U(H) 1.2 to 1.5 times *U*_eq_(C). The amino and hydroxy H-atoms were located in a difference Fourier map, and were refined with distance restraints (N–H 0.88±0.01, O–H 0.84±0.01 Å); their temperature factors were freely refined.

### Crystal data

**Table:**

C_15_H_16_N_2_O_3_S	*Z* = 4
*M**_r_* = 304.36	*F*_000_ = 640
Triclinic, *P*1	*D*_x_ = 1.415 Mg m^−^^3^
Hall symbol: -P 1	Mo *K*α radiation λ = 0.71073 Å
*a* = 5.1547 (1) Å	Cell parameters from 7775 reflections
*b* = 17.0321 (2) Å	θ = 2.2–30.4º
*c* = 18.2635 (1) Å	µ = 0.24 mm^−^^1^
α = 63.192 (1)º	*T* = 100 (2) K
β = 88.577 (1)º	Block, colorless
γ = 86.345 (1)º	0.18 × 0.14 × 0.06 mm
*V* = 1428.19 (4) Å^3^	

### Data collection

**Table:**

Bruker SMART APEX diffractometer	6415 independent reflections
Radiation source: fine-focus sealed tube	5603 reflections with *I* > 2σ(*I*)
Monochromator: graphite	*R*_int_ = 0.018
*T* = 100(2) K	θ_max_ = 27.5º
ω scans	θ_min_ = 1.3º
Absorption correction: Multi-scan(SADABS; Sheldrick, 1996)	*h* = −6→6
*T*_min_ = 0.958, *T*_max_ = 0.986	*k* = −22→22
12657 measured reflections	*l* = −23→22

### Refinement

**Table:**

Refinement on *F*^2^	Secondary atom site location: difference Fourier map
Least-squares matrix: full	Hydrogen site location: inferred from neighbouring sites
*R*[*F*^2^ > 2σ(*F*^2^)] = 0.042	H atoms treated by a mixture of independent and constrained refinement
*wR*(*F*^2^) = 0.132	*w* = 1/[σ^2^(*F*_o_^2^) + (0.0741*P*)^2^ + 1.3*P*] where *P* = (*F*_o_^2^ + 2*F*_c_^2^)/3
*S* = 1.05	(Δ/σ)_max_ = 0.001
6415 reflections	Δρ_max_ = 0.54 e Å^−^^3^
399 parameters	Δρ_min_ = −0.58 e Å^−^^3^
4 restraints	Extinction correction: none
Primary atom site location: structure-invariant direct methods	

### Fractional atomic coordinates and isotropic or equivalent isotropic displacement parameters (Å^2^)

**Table:**

	*x*	*y*	*z*	*U*_iso_*/*U*_eq_	
S1	1.00270 (8)	0.59346 (3)	0.36837 (3)	0.01389 (12)	
S2	0.23309 (8)	0.87206 (3)	0.94073 (3)	0.01512 (12)	
O1	1.1834 (3)	0.79133 (9)	0.43334 (9)	0.0198 (3)	
O2	1.2673 (3)	0.59821 (9)	0.38815 (8)	0.0181 (3)	
O3	0.9214 (3)	0.51803 (9)	0.36199 (9)	0.0190 (3)	
O4	0.1038 (3)	0.73122 (10)	0.80833 (9)	0.0238 (3)	
O5	−0.0223 (3)	0.84056 (10)	0.94695 (9)	0.0206 (3)	
O6	0.3106 (3)	0.90879 (9)	0.99282 (9)	0.0196 (3)	
N1	0.8800 (3)	0.66835 (10)	0.45822 (10)	0.0153 (3)	
N2	0.8209 (3)	0.59890 (11)	0.44147 (10)	0.0150 (3)	
N3	0.3956 (3)	0.74366 (11)	0.91380 (10)	0.0163 (3)	
N4	0.4405 (3)	0.78638 (11)	0.96235 (10)	0.0159 (3)	
C1	1.0427 (4)	0.80153 (13)	0.49302 (12)	0.0165 (4)	
C2	1.1112 (4)	0.86743 (13)	0.51253 (13)	0.0200 (4)	
H2	1.2525	0.9023	0.4851	0.024*	
C3	0.9752 (4)	0.88270 (13)	0.57162 (13)	0.0205 (4)	
H3	1.0244	0.9282	0.5840	0.025*	
C4	0.7677 (4)	0.83279 (13)	0.61327 (12)	0.0189 (4)	
C5	0.7016 (4)	0.76637 (13)	0.59405 (12)	0.0173 (4)	
H5	0.5617	0.7313	0.6225	0.021*	
C6	0.8335 (4)	0.74898 (12)	0.53418 (11)	0.0151 (4)	
C7	0.6197 (4)	0.84927 (15)	0.67793 (14)	0.0243 (4)	
H7A	0.4382	0.8351	0.6783	0.037*	
H7B	0.6264	0.9114	0.6656	0.037*	
H7C	0.6985	0.8120	0.7318	0.037*	
C8	0.7526 (4)	0.67782 (12)	0.51600 (11)	0.0152 (4)	
C9	0.5429 (4)	0.61962 (13)	0.56463 (13)	0.0208 (4)	
H9A	0.4909	0.5847	0.5376	0.031*	
H9B	0.3926	0.6559	0.5682	0.031*	
H9C	0.6077	0.5800	0.6199	0.031*	
C10	0.9242 (4)	0.68803 (12)	0.27615 (11)	0.0153 (4)	
C11	0.7132 (4)	0.68724 (14)	0.23050 (12)	0.0200 (4)	
H11	0.6139	0.6367	0.2489	0.024*	
C12	0.6499 (4)	0.76133 (14)	0.15765 (12)	0.0219 (4)	
H12	0.5065	0.7616	0.1258	0.026*	
C13	0.7956 (4)	0.83520 (14)	0.13098 (12)	0.0224 (4)	
H13	0.7512	0.8859	0.0811	0.027*	
C14	1.0053 (4)	0.83500 (14)	0.17703 (13)	0.0242 (4)	
H14	1.1050	0.8855	0.1584	0.029*	
C15	1.0708 (4)	0.76134 (14)	0.25038 (12)	0.0206 (4)	
H15	1.2136	0.7612	0.2823	0.025*	
C16	0.2653 (4)	0.66099 (14)	0.81883 (12)	0.0194 (4)	
C17	0.2155 (4)	0.61764 (15)	0.77206 (13)	0.0230 (4)	
H17	0.0730	0.6380	0.7348	0.028*	
C18	0.3706 (4)	0.54571 (14)	0.77928 (13)	0.0222 (4)	
H18	0.3328	0.5170	0.7470	0.027*	
C19	0.5829 (4)	0.51392 (14)	0.83316 (13)	0.0209 (4)	
C20	0.6317 (4)	0.55738 (13)	0.87953 (12)	0.0189 (4)	
H20	0.7759	0.5367	0.9161	0.023*	
C21	0.4775 (4)	0.63059 (13)	0.87480 (12)	0.0171 (4)	
C22	0.7530 (4)	0.43515 (15)	0.84084 (15)	0.0273 (5)	
H22A	0.9231	0.4364	0.8630	0.041*	
H22B	0.7753	0.4369	0.7867	0.041*	
H22C	0.6709	0.3810	0.8779	0.041*	
C23	0.5394 (4)	0.67411 (13)	0.92575 (11)	0.0162 (4)	
C24	0.7550 (4)	0.63615 (13)	0.98790 (12)	0.0196 (4)	
H24A	0.7488	0.6660	1.0229	0.029*	
H24B	0.9226	0.6446	0.9596	0.029*	
H24C	0.7344	0.5731	1.0217	0.029*	
C25	0.2788 (4)	0.94842 (12)	0.83776 (11)	0.0157 (4)	
C26	0.4827 (4)	1.00410 (13)	0.81963 (13)	0.0202 (4)	
H26	0.5954	1.0000	0.8617	0.024*	
C27	0.5177 (4)	1.06564 (14)	0.73883 (13)	0.0225 (4)	
H27	0.6577	1.1034	0.7251	0.027*	
C28	0.3492 (4)	1.07231 (13)	0.67788 (13)	0.0210 (4)	
H28	0.3721	1.1154	0.6229	0.025*	
C29	0.1478 (4)	1.01618 (14)	0.69714 (13)	0.0215 (4)	
H29	0.0333	1.0210	0.6551	0.026*	
C30	0.1120 (4)	0.95307 (13)	0.77718 (12)	0.0185 (4)	
H30	−0.0240	0.9138	0.7903	0.022*	
H1O	1.110 (6)	0.7524 (18)	0.427 (2)	0.057 (10)*	
H4O	0.165 (6)	0.752 (2)	0.8385 (17)	0.043 (8)*	
H2N	0.657 (2)	0.5927 (17)	0.4337 (16)	0.025 (6)*	
H4N	0.600 (2)	0.8017 (16)	0.9621 (16)	0.022 (6)*	

### Atomic displacement parameters (Å^2^)

**Table:**

	*U*^11^	*U*^22^	*U*^33^	*U*^12^	*U*^13^	*U*^23^
S1	0.0143 (2)	0.0138 (2)	0.0141 (2)	−0.00056 (16)	−0.00083 (16)	−0.00670 (17)
S2	0.0133 (2)	0.0169 (2)	0.0150 (2)	−0.00203 (16)	−0.00012 (16)	−0.00687 (18)
O1	0.0216 (7)	0.0193 (7)	0.0200 (7)	−0.0062 (6)	0.0044 (5)	−0.0098 (6)
O2	0.0157 (6)	0.0183 (7)	0.0186 (7)	−0.0004 (5)	−0.0014 (5)	−0.0068 (6)
O3	0.0213 (7)	0.0173 (7)	0.0206 (7)	−0.0016 (5)	−0.0016 (5)	−0.0103 (6)
O4	0.0222 (7)	0.0268 (8)	0.0248 (8)	0.0032 (6)	−0.0080 (6)	−0.0140 (7)
O5	0.0145 (6)	0.0248 (7)	0.0205 (7)	−0.0042 (5)	0.0008 (5)	−0.0080 (6)
O6	0.0217 (7)	0.0225 (7)	0.0175 (7)	−0.0013 (6)	−0.0007 (5)	−0.0117 (6)
N1	0.0181 (8)	0.0142 (7)	0.0152 (7)	−0.0022 (6)	−0.0020 (6)	−0.0078 (6)
N2	0.0160 (7)	0.0160 (8)	0.0157 (7)	−0.0036 (6)	0.0015 (6)	−0.0091 (6)
N3	0.0169 (7)	0.0173 (8)	0.0162 (8)	−0.0034 (6)	−0.0004 (6)	−0.0084 (6)
N4	0.0152 (7)	0.0167 (8)	0.0166 (8)	−0.0014 (6)	−0.0026 (6)	−0.0080 (6)
C1	0.0182 (9)	0.0153 (9)	0.0140 (8)	0.0000 (7)	−0.0023 (7)	−0.0049 (7)
C2	0.0217 (9)	0.0157 (9)	0.0205 (9)	−0.0026 (7)	−0.0030 (7)	−0.0060 (8)
C3	0.0239 (10)	0.0169 (9)	0.0230 (10)	0.0006 (7)	−0.0065 (8)	−0.0110 (8)
C4	0.0207 (9)	0.0191 (9)	0.0175 (9)	0.0042 (7)	−0.0052 (7)	−0.0093 (8)
C5	0.0178 (9)	0.0168 (9)	0.0170 (9)	0.0005 (7)	−0.0023 (7)	−0.0074 (7)
C6	0.0182 (9)	0.0131 (8)	0.0132 (8)	0.0004 (7)	−0.0028 (7)	−0.0053 (7)
C7	0.0257 (10)	0.0265 (11)	0.0271 (11)	0.0034 (8)	−0.0021 (8)	−0.0181 (9)
C8	0.0164 (8)	0.0135 (8)	0.0132 (8)	0.0003 (7)	−0.0024 (7)	−0.0039 (7)
C9	0.0233 (10)	0.0181 (9)	0.0222 (10)	−0.0051 (8)	0.0081 (8)	−0.0102 (8)
C10	0.0165 (8)	0.0164 (9)	0.0127 (8)	−0.0003 (7)	0.0000 (7)	−0.0064 (7)
C11	0.0212 (9)	0.0202 (10)	0.0191 (9)	−0.0035 (7)	−0.0014 (7)	−0.0091 (8)
C12	0.0230 (10)	0.0260 (10)	0.0169 (9)	0.0012 (8)	−0.0050 (7)	−0.0101 (8)
C13	0.0272 (10)	0.0219 (10)	0.0145 (9)	0.0026 (8)	0.0006 (8)	−0.0057 (8)
C14	0.0290 (11)	0.0181 (10)	0.0225 (10)	−0.0055 (8)	−0.0002 (8)	−0.0061 (8)
C15	0.0217 (9)	0.0210 (10)	0.0189 (9)	−0.0029 (8)	−0.0018 (7)	−0.0086 (8)
C16	0.0186 (9)	0.0218 (10)	0.0176 (9)	−0.0035 (7)	0.0003 (7)	−0.0085 (8)
C17	0.0219 (10)	0.0283 (11)	0.0197 (10)	−0.0052 (8)	−0.0019 (8)	−0.0111 (9)
C18	0.0270 (10)	0.0257 (10)	0.0177 (9)	−0.0087 (8)	0.0045 (8)	−0.0124 (8)
C19	0.0213 (9)	0.0221 (10)	0.0208 (10)	−0.0073 (8)	0.0050 (8)	−0.0105 (8)
C20	0.0197 (9)	0.0197 (9)	0.0181 (9)	−0.0039 (7)	0.0011 (7)	−0.0091 (8)
C21	0.0177 (9)	0.0190 (9)	0.0157 (9)	−0.0050 (7)	0.0017 (7)	−0.0083 (8)
C22	0.0288 (11)	0.0269 (11)	0.0325 (12)	−0.0031 (9)	0.0015 (9)	−0.0188 (10)
C23	0.0161 (9)	0.0169 (9)	0.0138 (8)	−0.0040 (7)	0.0009 (7)	−0.0049 (7)
C24	0.0206 (9)	0.0177 (9)	0.0191 (9)	−0.0008 (7)	−0.0034 (7)	−0.0070 (8)
C25	0.0167 (9)	0.0146 (8)	0.0136 (8)	0.0002 (7)	0.0003 (7)	−0.0047 (7)
C26	0.0182 (9)	0.0200 (9)	0.0209 (10)	−0.0019 (7)	−0.0025 (7)	−0.0075 (8)
C27	0.0191 (9)	0.0174 (9)	0.0262 (11)	−0.0029 (7)	0.0015 (8)	−0.0054 (8)
C28	0.0235 (10)	0.0184 (9)	0.0180 (9)	0.0028 (8)	0.0017 (7)	−0.0060 (8)
C29	0.0243 (10)	0.0233 (10)	0.0188 (9)	0.0019 (8)	−0.0038 (8)	−0.0115 (8)
C30	0.0184 (9)	0.0195 (9)	0.0192 (9)	−0.0012 (7)	−0.0018 (7)	−0.0099 (8)

### Geometric parameters (Å, °)

**Table:**

S1—O3	1.4303 (14)	C11—C12	1.387 (3)
S1—O2	1.4360 (14)	C11—H11	0.9500
S1—N2	1.6455 (16)	C12—C13	1.390 (3)
S1—C10	1.7603 (19)	C12—H12	0.9500
S2—O6	1.4296 (14)	C13—C14	1.385 (3)
S2—O5	1.4355 (14)	C13—H13	0.9500
S2—N4	1.6526 (17)	C14—C15	1.391 (3)
S2—C25	1.7591 (19)	C14—H14	0.9500
O1—C1	1.364 (2)	C15—H15	0.9500
O1—H1O	0.84 (3)	C16—C17	1.394 (3)
O4—C16	1.355 (2)	C16—C21	1.419 (3)
O4—H4O	0.85 (3)	C17—C18	1.376 (3)
N1—C8	1.295 (2)	C17—H17	0.9500
N1—N2	1.401 (2)	C18—C19	1.399 (3)
N2—H2N	0.879 (10)	C18—H18	0.9500
N3—C23	1.290 (3)	C19—C20	1.389 (3)
N3—N4	1.408 (2)	C19—C22	1.508 (3)
N4—H4N	0.877 (10)	C20—C21	1.405 (3)
C1—C2	1.389 (3)	C20—H20	0.9500
C1—C6	1.416 (3)	C21—C23	1.478 (3)
C2—C3	1.384 (3)	C22—H22A	0.9800
C2—H2	0.9500	C22—H22B	0.9800
C3—C4	1.392 (3)	C22—H22C	0.9800
C3—H3	0.9500	C23—C24	1.500 (3)
C4—C5	1.390 (3)	C24—H24A	0.9800
C4—C7	1.511 (3)	C24—H24B	0.9800
C5—C6	1.406 (3)	C24—H24C	0.9800
C5—H5	0.9500	C25—C30	1.389 (3)
C6—C8	1.479 (2)	C25—C26	1.393 (3)
C7—H7A	0.9800	C26—C27	1.387 (3)
C7—H7B	0.9800	C26—H26	0.9500
C7—H7C	0.9800	C27—C28	1.388 (3)
C8—C9	1.495 (3)	C27—H27	0.9500
C9—H9A	0.9800	C28—C29	1.386 (3)
C9—H9B	0.9800	C28—H28	0.9500
C9—H9C	0.9800	C29—C30	1.386 (3)
C10—C15	1.387 (3)	C29—H29	0.9500
C10—C11	1.391 (3)	C30—H30	0.9500
			
O3—S1—O2	120.83 (8)	C11—C12—H12	119.9
O3—S1—N2	104.70 (8)	C13—C12—H12	119.9
O2—S1—N2	106.84 (8)	C14—C13—C12	120.07 (19)
O3—S1—C10	108.61 (9)	C14—C13—H13	120.0
O2—S1—C10	107.30 (9)	C12—C13—H13	120.0
N2—S1—C10	107.97 (9)	C13—C14—C15	120.38 (19)
O6—S2—O5	120.70 (9)	C13—C14—H14	119.8
O6—S2—N4	104.46 (8)	C15—C14—H14	119.8
O5—S2—N4	106.50 (9)	C10—C15—C14	118.93 (18)
O6—S2—C25	109.14 (9)	C10—C15—H15	120.5
O5—S2—C25	107.40 (9)	C14—C15—H15	120.5
N4—S2—C25	108.03 (9)	O4—C16—C17	117.22 (18)
C1—O1—H1O	104 (3)	O4—C16—C21	122.92 (17)
C16—O4—H4O	105 (2)	C17—C16—C21	119.85 (19)
C8—N1—N2	118.12 (16)	C18—C17—C16	120.71 (19)
N1—N2—S1	112.74 (12)	C18—C17—H17	119.6
N1—N2—H2N	118.6 (17)	C16—C17—H17	119.6
S1—N2—H2N	110.1 (17)	C17—C18—C19	121.38 (18)
C23—N3—N4	118.41 (16)	C17—C18—H18	119.3
N3—N4—S2	111.80 (12)	C19—C18—H18	119.3
N3—N4—H4N	115.8 (17)	C20—C19—C18	117.66 (19)
S2—N4—H4N	110.0 (17)	C20—C19—C22	121.06 (19)
O1—C1—C2	117.07 (17)	C18—C19—C22	121.29 (18)
O1—C1—C6	123.00 (17)	C19—C20—C21	123.01 (18)
C2—C1—C6	119.92 (18)	C19—C20—H20	118.5
C3—C2—C1	120.50 (19)	C21—C20—H20	118.5
C3—C2—H2	119.8	C20—C21—C16	117.39 (17)
C1—C2—H2	119.8	C20—C21—C23	120.48 (17)
C2—C3—C4	121.39 (18)	C16—C21—C23	122.13 (18)
C2—C3—H3	119.3	C19—C22—H22A	109.5
C4—C3—H3	119.3	C19—C22—H22B	109.5
C5—C4—C3	117.86 (18)	H22A—C22—H22B	109.5
C5—C4—C7	120.74 (19)	C19—C22—H22C	109.5
C3—C4—C7	121.40 (18)	H22A—C22—H22C	109.5
C4—C5—C6	122.59 (19)	H22B—C22—H22C	109.5
C4—C5—H5	118.7	N3—C23—C21	115.90 (17)
C6—C5—H5	118.7	N3—C23—C24	123.93 (17)
C5—C6—C1	117.73 (17)	C21—C23—C24	120.16 (17)
C5—C6—C8	120.05 (17)	C23—C24—H24A	109.5
C1—C6—C8	122.22 (17)	C23—C24—H24B	109.5
C4—C7—H7A	109.5	H24A—C24—H24B	109.5
C4—C7—H7B	109.5	C23—C24—H24C	109.5
H7A—C7—H7B	109.5	H24A—C24—H24C	109.5
C4—C7—H7C	109.5	H24B—C24—H24C	109.5
H7A—C7—H7C	109.5	C30—C25—C26	121.68 (18)
H7B—C7—H7C	109.5	C30—C25—S2	120.18 (15)
N1—C8—C6	115.73 (17)	C26—C25—S2	118.14 (15)
N1—C8—C9	123.48 (17)	C27—C26—C25	118.62 (18)
C6—C8—C9	120.75 (16)	C27—C26—H26	120.7
C8—C9—H9A	109.5	C25—C26—H26	120.7
C8—C9—H9B	109.5	C26—C27—C28	120.36 (19)
H9A—C9—H9B	109.5	C26—C27—H27	119.8
C8—C9—H9C	109.5	C28—C27—H27	119.8
H9A—C9—H9C	109.5	C29—C28—C27	120.14 (19)
H9B—C9—H9C	109.5	C29—C28—H28	119.9
C15—C10—C11	121.32 (18)	C27—C28—H28	119.9
C15—C10—S1	120.15 (14)	C30—C29—C28	120.52 (19)
C11—C10—S1	118.53 (15)	C30—C29—H29	119.7
C12—C11—C10	119.01 (19)	C28—C29—H29	119.7
C12—C11—H11	120.5	C29—C30—C25	118.66 (18)
C10—C11—H11	120.5	C29—C30—H30	120.7
C11—C12—C13	120.27 (18)	C25—C30—H30	120.7
			
C8—N1—N2—S1	−178.55 (13)	C12—C13—C14—C15	−0.4 (3)
O3—S1—N2—N1	178.10 (12)	C11—C10—C15—C14	−0.5 (3)
O2—S1—N2—N1	48.83 (14)	S1—C10—C15—C14	179.65 (16)
C10—S1—N2—N1	−66.31 (14)	C13—C14—C15—C10	0.6 (3)
C23—N3—N4—S2	−178.21 (14)	O4—C16—C17—C18	−179.93 (19)
O6—S2—N4—N3	−178.19 (12)	C21—C16—C17—C18	−0.3 (3)
O5—S2—N4—N3	53.04 (14)	C16—C17—C18—C19	−0.3 (3)
C25—S2—N4—N3	−62.08 (14)	C17—C18—C19—C20	0.2 (3)
O1—C1—C2—C3	179.06 (17)	C17—C18—C19—C22	−179.9 (2)
C6—C1—C2—C3	−0.5 (3)	C18—C19—C20—C21	0.4 (3)
C1—C2—C3—C4	0.2 (3)	C22—C19—C20—C21	−179.54 (19)
C2—C3—C4—C5	0.4 (3)	C19—C20—C21—C16	−0.9 (3)
C2—C3—C4—C7	179.82 (18)	C19—C20—C21—C23	−179.97 (18)
C3—C4—C5—C6	−0.8 (3)	O4—C16—C21—C20	−179.57 (18)
C7—C4—C5—C6	179.81 (18)	C17—C16—C21—C20	0.8 (3)
C4—C5—C6—C1	0.5 (3)	O4—C16—C21—C23	−0.5 (3)
C4—C5—C6—C8	−179.50 (17)	C17—C16—C21—C23	179.88 (18)
O1—C1—C6—C5	−179.38 (17)	N4—N3—C23—C21	178.11 (16)
C2—C1—C6—C5	0.1 (3)	N4—N3—C23—C24	−0.7 (3)
O1—C1—C6—C8	0.6 (3)	C20—C21—C23—N3	176.27 (18)
C2—C1—C6—C8	−179.87 (17)	C16—C21—C23—N3	−2.8 (3)
N2—N1—C8—C6	176.62 (15)	C20—C21—C23—C24	−4.9 (3)
N2—N1—C8—C9	−1.1 (3)	C16—C21—C23—C24	176.08 (18)
C5—C6—C8—N1	177.95 (17)	O6—S2—C25—C30	−147.06 (15)
C1—C6—C8—N1	−2.0 (3)	O5—S2—C25—C30	−14.60 (18)
C5—C6—C8—C9	−4.2 (3)	N4—S2—C25—C30	99.92 (16)
C1—C6—C8—C9	175.76 (18)	O6—S2—C25—C26	31.95 (18)
O3—S1—C10—C15	−151.83 (16)	O5—S2—C25—C26	164.42 (15)
O2—S1—C10—C15	−19.67 (18)	N4—S2—C25—C26	−81.06 (17)
N2—S1—C10—C15	95.17 (17)	C30—C25—C26—C27	0.1 (3)
O3—S1—C10—C11	28.32 (18)	S2—C25—C26—C27	−178.86 (16)
O2—S1—C10—C11	160.48 (15)	C25—C26—C27—C28	1.2 (3)
N2—S1—C10—C11	−84.68 (16)	C26—C27—C28—C29	−1.3 (3)
C15—C10—C11—C12	0.3 (3)	C27—C28—C29—C30	0.1 (3)
S1—C10—C11—C12	−179.87 (15)	C28—C29—C30—C25	1.2 (3)
C10—C11—C12—C13	−0.1 (3)	C26—C25—C30—C29	−1.3 (3)
C11—C12—C13—C14	0.2 (3)	S2—C25—C30—C29	177.65 (15)

### Hydrogen-bond geometry (Å, °)

**Table:**

*D*—H···*A*	*D*—H	H···*A*	*D*···*A*	*D*—H···*A*
O1—H1o···N1	0.84 (3)	1.80 (2)	2.562 (2)	151 (4)
O4—H4o···N3	0.85 (3)	1.79 (2)	2.563 (2)	150 (3)
N2—H2n···O2^i^	0.88 (1)	2.18 (1)	3.040 (2)	168 (2)
N4—H4n···O5^ii^	0.88 (1)	2.07 (1)	2.942 (2)	173 (2)

Symmetry codes: (i) *x*−1, *y*, *z*; (ii) *x*+1, *y*, *z*.
